# Liver Resection: To Drain or not to Drain?

**DOI:** 10.1155/1998/12974

**Published:** 1998

**Authors:** David L. Morris

**Affiliations:** Department of Surgery The St George Hospital Sydney NSW 2217 Australia

## Abstract

*Purpose*: A prospective, randomized trial was performed to determine if intra-abdominal drainage catheters are necessary after elective liver resection.

*Patients and Methods*: Between April 1992 and April 1994, 120 patients subjected to liver resection, stratified by extent of resection and by surgeon, were randomized to receive or not receive operative closed-suction drainage. Operative blood loss was not an exclusion criteria, and no patient who consented to the study was excluded.

*Results*: Eighty-seven patients (73%) had resection of one hepatic lobe or more (27 lobectomies, 54 trisegmentectomies, and 6 bilobar atypical resections) and 33 had less than a lobectomy (8 wedge resections or enucleations, 9 segmentectomies, and 16 bisegmentectomies). Eighty-four patients (70%) had metastatic cancer and 36 patients (30%) had primary liver pathology. There were no differences in outcome, including length of hospital stay (no drain, 13.4 ± 0.9 days; drain, 13.1 ± 0.8 days; P not significant [NS]), mortality (no drain, 3.3%; drain, 3.3%), complication rate (no drain, 43%; drain, 48%; *n*= NS), or requirement for subsequent percutaneous drainage (no drain, 18%; drain, 8%; *P*= NS). All infected collections (*n*= 3) occured in operatively drained patients. Two other complications were directly related to the operatively placed drains. One patient developed a subcutaneous abscess at the drain site, and a second developed a subcutaneous drain tract tumor recurrence as the only current site of recurrence.

*Conclusion*: In the first 50 consecutive resections performed since the conclusion of this trial, only 4 patients (8%) have required subsequent percutaneous drainage. We conclude that abdominal drainage is unnecessary after elective liver resection,


*HPB Surgery*, 1998, Vol. 10, pp. 405–418
Reprints available directly from the publisher
Photocopying permitted by license only

© 1998 OPA (Overseas Publishers Association)
Amsterdam B.V. Published under license
under the Harwood Academic Publishers imprint,
part of The Gordon and Breach Publishing Group.
Printed in India.

**HPB INTERNATIONAL**

**EDITORIAL & ABSTRACTING SERVICE**

JOHN TERBLANCHE EDITOR
Department of Surgery,
Medical School · Observatory 7925
Cape Town · South Africa
Telephone: 27-21-406-6204
Telefax 27-21-448-6461
E-mail: jterblan@uctgsh1.uct.ac.za

### Liver Resection: To Drain or not to Drain?

### ABSTRACT

*Fong, Y., Brennan, M. F., Brown, K., Heffernan, N., Blumgart, L. H, (1996) Drainage is unnecessary after elective liver resection, The American Journal of Surgery, 171, 158–162.*

***Purpose:*** **A prospective, randomized trial was performed to determine if intra-abdominal drainage catheters are necessary after elective liver resection.**
***Patients and Methods:*** **Between April 1992 and April 1994, 120 patients subjected to liver resection, stratified by extent of resection and by surgeon, were randomized to receive or not receive operative closed-suction drainage. Operative blood loss was not an exclusion criteria, and no patient who consented to the study was excluded.**
***Results:*** **Eighty-seven patients (73%) had resection of one hepatic lobe or more (27 lobectomies, 54 trisegmentectomies, and 6 bilobar atypical resections) and 33 had less than a lobectomy (8 wedge resections or enucleations, 9 segmentectomies, and 16 bisegmentectomies). Eighty-four patients (70%) had metastatic cancer and 36 patients (30%) had primary liver pathology. There were no differences in outcome, including length of hospital stay (no drain, 13.4 ± 0.9 days; drain, 13.1 ± 0.8 days; *P* = not significant [NS]), mortality (no drain, 3.3%; drain, 3.3%), complication rate (no drain, 43%; drain, 48%; *P* = NS), or requirement for subsequent percutaneous drainage (no drain, 18%; drain, 8%; *P* = NS). All infected collections (*n* = 3) occured in operatively drained patients. Two other complications were directly related to the operatively placed drains. One patient developed a subcutaneous abscess at the drain site, and a second developed a subcutaneous drain tract tumor recurrence as the only current site of recurrence.**
***Conclusion:*** **In the first 50 consecutive resections performed since the conclusion of this trial, only 4 patients (8%) have required subsequent percutaneous drainage. We conclude that abdominal drainage is unnecessary after elective liver resection, *Am J Surg.*, 1996, 171, 158–162.**

*Keywords:* Liver resection, abdominal drainage

### PAPER DISCUSSION

This paper concerns 120 patients undergoing liver resection, including 87 undergoing lobectomy or more. Patients were randomized intraoperatively – which is in itself not an optimal design and details of all excluded patients are not included.

Patients were randomly allocated to closed suction drains (Jackson-Pratt) or controls. No significant difference in death (3.3%), hospital stay (13 days), biliary fistula (5%). Five operatively drained patients required peritoneal drainage whereas 11 in the undrained group (18%)

required percutaneous drainage – none of them were infected whereas 3 of the drain group collections were infected.

The paper concludes that drainage after liver resection is unnecessary. I have several problems with this:

1. The power of this study to detect adverse outcome in the no drain group is very limited – with approximately 50 patients in each group, if a complication which occured with a rate of 5% in the drained group was three times more frequent in the undrained group this would *not* be detected.
2. The study does not show any advantage to not draining. In fact, there was significantly higher need for percutaneous post-op drainage.
3. Whilst two complications of drainage were seen – drain site abscess and drain site cancer recurrence, ie. the former would seem a relatively minor complication and even if the drain site appeared to be the only site of recurrence in the second patient, it would seem likely that if enough viable tumour cells were transplanted into the drain site that they would have developed at other sites in time.

David L. Morris
Professor of Surgery
Department of Surgery
The St George Hospital
Sydney NSW 2217 – Australia

### Is Chemoembolisation of Value in Inoperable Primary Hepatocellular Carcinoma

### ABSTRACT

*Ryder, S. D., Rizzi, P. M., Metivier, E., Karani, J. and Williams, R. (1996) Chemoembolisation with lipiodol and doxorubicin: applicability in British patients with hepatocellular carcinoma, Gut, 38, 125–128.*

**Chemoembolisation has been extensively used as primary treatment for unresectable hepatocellular carcinoma (HCC). In this unit, 185 patients with a new diagnosis of HCC not amenable to surgery were seen between 1988 and 1991. Intended therapy for these patients was chemoembolisation with doxorubicin (60 mg/m$^2$) and lipiodol, repeated at six week intervals until it was technically no longer possible or until complete tumour response had been obtained. Chemoembolisation was possible in 67 of the 185 (37%). Reasons for exclusion were portal vein occlusion (*n* = 36), decompensated cirrhosis (*n* = 44), distant metastases (*n* = 5), diffuse tumour or unsuitable anatomy (tumour or vasculature) (*n* = 11), patient refusal (*n* = 11), and other (*n* = 11). Patients excluded from treatment survived for a median of 10 weeks (range 3 days-19 months). In patients treated, 18 had small HCC ( 4 cm) and 49 had large or multifocal HCC. Chemoembolisation was carried out a median of two sessions for small and three sessions for large tumours. Ten of 18 patients with small HCC showed a 50% or greater reduction in tumour size. Five of 49 patients with large or multifocal tumours showed a response to treatment. Median overall survival for treated patients was 36 weeks (range 3 days–4 years). One patient has subsequently undergone liver transplantation with no recurrence and minimal residual disease at transplantation. Two other patients are alive three years after chemoembolisation, one with no evidence of recurrent disease. No patient was thought suitable for surgery after their response to chemoembolisation. Chemotherapy related complications were seen in 22%. Complications were significantly more common in patients with larger tumours and poor liver reserve. Five patients died as a result of chemotherapy related complications. In conclusion, only one third of UK patients with unresectable HCC are treatable by chemoembolisation. Results with small**

